# Elevational diversity gradients of Tibetan loaches: The relative roles of ecological and evolutionary processes

**DOI:** 10.1002/ece3.3504

**Published:** 2017-10-22

**Authors:** Chenguang Feng, Yongjie Wu, Fei Tian, Chao Tong, Yongtao Tang, Renyi Zhang, Guogang Li, Kai Zhao

**Affiliations:** ^1^ Key Laboratory of Adaptation and Evolution of Plateau Biota, and Laboratory of Plateau Fish Evolutionary and Functional Genomics Northwest Institute of Plateau Biology Chinese Academy of Sciences Xining Qinghai China; ^2^ University of Chinese Academy of Sciences Beijing China; ^3^ Key Laboratory of Bio‐resources and Eco‐environment of Ministry of Education College of Life Sciences Sichuan University Chengdu Sichuan China; ^4^ School of Life Sciences Guizhou Normal University Guiyang Guizhou China; ^5^ Center for Integrative Conservation Xishuangbanna Tropical Botanical Garden Chinese Academy of Sciences Mengla Yunnan China

**Keywords:** biogeography, elevational gradients, evolution, species richness, Tibetan Plateau, *Triplophysa*

## Abstract

It is widely believed that species richness patterns (SRPs) are shaped by both ecological and evolutionary processes. However, the relative roles of these processes remain unclear, especially for aquatic organisms. In this study, we integrated ecological and evolutionary measures to tease apart the relative influences of these factors on the SRP of Tibetan loaches along an extensive elevational gradient. We found that the Tibetan loaches displayed a richness pattern that peaked at midelevations. The mean annual temperature (MAT), mid‐domain effect (MDE), and summed age of colonization (SAC, complex of colonization age and colonization frequency) were the main drivers, accounting for 85%, 51%, and 88% of the variations in the SRP, respectively. The three predictors had very high combined effects (MAT‐MDE‐SAC, MAT‐SAC, and MDE‐SAC were 44%, 38%, and 6%, respectively). Our analyses suggested that energy input, time‐for‐speciation, and species dispersal may directly guide the SRP or mediate it by geometric constraints. Conclusively, the SRP of the Tibetan loaches with elevation is the outcome of interactions between biogeographical processes and regional ecological conditions.

## 1 INTRODUCTION

The mechanism of geographic variation in species richness is a fundamental issue in ecology (Kozak & Wiens, 2010), and it has been explored by ecologists for decades (Sanders & Rahbek, 2012). Compared to latitudinal gradients, studies on elevational gradients have played prominent roles in advancing our knowledge of the causes of species richness variations (Graham et al., 2014; Grytnes & McCain, 2007; Sanders & Rahbek, 2012). Species richness patterns (SRPs) along elevational gradients generally present three forms: decreasing diversity with increasing elevation, high diversity at low‐elevation plateaus, and unimodal richness patterns peaking at midelevations (McCain, 2009, 2010; Rahbek, 2005). Ecologists have attempted to understand the causes of SRPs from various aspects. All interpretations can be classified into ecological (climatic hypotheses and spatial hypotheses) and evolutionary (evolutionary hypotheses and historical hypotheses) factors (Kozak & Wiens, 2010; Li et al., 2009; McCain, 2007a,b; Wiens, Parra‐Olea, García‐París, & Wake, 2007). Specifically, the climatic hypotheses propose that SRPs are generated by the variations in water and energy (Lomolino, 2001). Spatial hypotheses comprise species‐area relationships and the mid‐domain effect (MDE). The former argues that larger areas harbor more species (Rahbek, 1997), while the MDE indicates that only dispersal limitations on either side of the altitudinal gradient generate more overlap at midelevations and create hump‐shaped richness patterns (Colwell & Hurtt, 1994; Colwell & Lees, 2000). Evolutionary hypotheses (e.g., “montane species pump” hypothesis) claim that high species diversity is generated by a high net diversification rate (Smith, De Oca, Reeder, & Wiens, 2007). Historical hypotheses (e.g., “montane museum” hypothesis) assert that longer colonization times enable more species to establish and accumulate (Stephens & Wiens, 2003).

It is generally believed that SRPs are shaped by both ecological and evolutionary processes (Graham et al., 2014; Kozak & Wiens, 2010; Sanders & Rahbek, 2012; Wu et al., 2014). However, the effects of ecological and evolutionary processes on SRPs have often been tested separately. The few studies that have considered both processes stated only the combined effects (Kozak & Wiens, 2012; Li et al., 2009; Wu et al., 2014). The relative roles of ecological and evolutionary processes in producing SRPs remain unclear.

Tibetan loaches (Cobitoidea: *Triplophysa*) are important components of the ichthyofauna on the Tibetan Plateau (Chen, Chen, & Liu, 1996; Wu & Wu, 1992; Zhu, 1989). The northeastern margin of the Tibetan Plateau has many climatic zones that span the elevational gradients, and the Tibetan loaches in this area stem from the same species pool and have undergone similar evolutionary histories (Feng et al., 2017; He, Chen, & Chen, 2006). Thus, Tibetan loaches on the northeastern margin of the Tibetan Plateau are an ideal model to examine the mechanisms underlying the SRPs (see review in Graham et al., 2014). In this study, we integrate ecological and evolutionary approaches to examine the causes of the SRPs of Tibetan loaches on the northeastern margin of the Tibetan Plateau. Moreover, many studies have found potential links between SRPs and Rapoport's rule (Brehm, Colwell, & Kluge, 2007; Fleishman, Austin, & Weiss, 1998; Sanders, 2002). Therefore, we also tested Rapoport's rule (Stevens, 1992) and the climatic variability hypothesis (Gaston & Chown, 1999) to determine the influence of climatic variability on the ranges of species. Based on those comprehensive means, we aimed to (1) depict the species richness or distribution patterns of Tibetan loaches along altitudinal gradients and (2) examine the different factors explaining the observed SRP.

## 2 MATERIALS AND METHODS

### 2.1 Data collection

**Figure 1:**
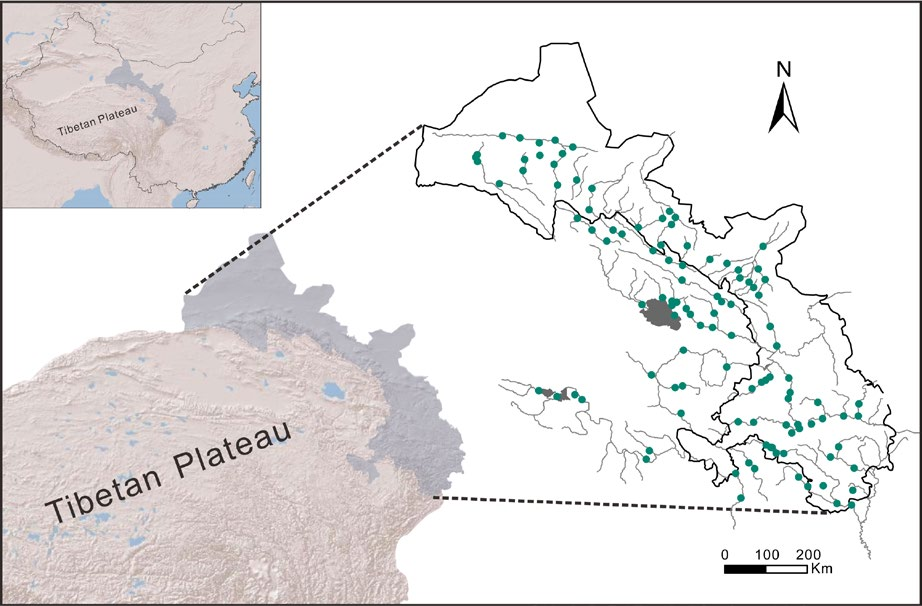
Graphical representation of the sampling sites in our field surveys and drainage system. The grassy dots are the sampling sites. The gray and blue patches and the gray lines depict the drainage system

The study area is in the northeastern margin of the Tibetan Plateau (32°14′–40°36′N, 94°19′–106°08′E) with a total area of approximately 469,000 km^2^ (Figure 1). The altitudes of the drainage systems in this region range from 600 to 4,300 m (a.s.l.), and the systems were treated as a single unit as they were historically connected (Chen et al., 1996; Wu & Wu, 1992; Zhu, 1989). Nineteen valid species have been detected in this region (Feng et al., 2017).

We compiled information on the distribution and altitude of Tibetan loaches from the literature and field surveys (see Appendices S1, S2; Table A1 and Fig. A1 in Appendix S3). We performed a rarefaction analysis in EstimateS ver. 8.2 (Colwell, 2009) to assess the adequacy of the sampling. The species range was ascertained by interpolating the elevational limitation values (see Appendix S1). We divided the entire region into 19 bands (200 m for each elevational band) and calculated the species richness based on the interpolated ranges (the number of species with ranges in each band was counted). Moreover, we also calculated the observed species richness using the number of species detected in each elevational band in our field surveys.

### 2.2 Ecological factors: climate, area, and MDE

We obtained climate and area data from the WorldClim database ( http://www.worldclim.org) in 30‐arc‐second (*c*. 1 km^2^) digital maps, including elevation, mean annual temperature (MAT), mean diurnal range (MDR), temperature seasonality (TS), annual precipitation (AP), precipitation seasonality (PS), and precipitation of the driest quarter (PDQ; see Fig. A2 in Appendix S3). As the effective area for fish is not equal to the total land area in each band, we used the drainage systems as a mask to extract the corresponding water areas (see Fig. A3 in Appendix S3). We used river basin area (rivers without lakes) and total drainage area (rivers and lakes) to denote the area in each band because lakes cannot represent the environmental heterogeneity as much as the water areas they possess. The data extraction was implemented in ArcGIS 10.2 (ESRI, CA, USA). The data handling followed the method described by Li et al. (2009).

We utilized the MDE model in RangeModel ver. 5 (Colwell, 2006) to test the MDE. We conducted 50,000 simulations of random range placement within the bounded domain of Tibetan loaches as described in Box 5 of Colwell and Lees (2000). The computed mean richness and its 95% confidence interval were used to evaluate the explanatory power of the MDE on the SRP.

### 2.3 Evolutionary factors: diversification rates and timing of colonization

We first developed the time‐calibrated phylogeny of Tibetan loaches on the northeastern margin of the Tibetan Plateau (see Appendix S1; Table A2, Figs. A4‐A6 in Appendix S3). Based on the maximum likelihood (ML) phylogenetic tree, the ancestral elevational distributions were reconstructed using the range sizes and midpoints of extant species in Mesquite ver. 2.75 (Maddison & Maddison, 2001) according to the method described by Wu et al. (2014) (see Fig. A7 in Appendix S3).

We used the shift of diversification rates along elevations to test the “montane species pump” hypothesis (Wiens, Sukumaran, Pyron, & Brown, 2009; Wiens et al., 2007). The diversification rates of each clade for both the stem group and the crown group were calculated using the method of moments estimator (Magallon & Sanderson, 2001). We assumed three values for the relative extinction rate (*e *=* *0, 0.45 and 0.9) that was unknown but required by the calculation (see Table A3 in Appendix S3). The analyses were performed using the “geiger” package (Harmon, Weir, Brock, Glor, & Challenger, 2008) in R v3.2.3 ( http://www.R-project.org). We also applied another new model, Bayesian analysis of macroevolutionary mixtures (BAMM, www.bammproject.org), to inspect the “montane species pump” hypothesis, which enabled the reconstruction of the dynamic diversification process through time and among lineages without assuming a relative extinction rate (Rabosky, Donnellan, Grundler, & Lovette, 2014). We used BAMM v2.5.0 and the R package “BAMMtools” (Rabosky, Grundler, et al. 2014) to compute the clade‐specific mean rates (see Table A4 and Fig. A8 in Appendix S3). Both the predicted ancestral elevation and the mean elevation of each clade were used to evaluate the relationships of these factors with diversification rates.

To test the “montane museum” hypothesis, we calculated the oldest age, summed age, and average age of colonization as well as the colonization frequency in each elevational band following the colonization based on reconstructed range size and location (CRRL) method described by Wu et al. (2014) and tried to tease out which measures offer higher sensitives (see Table A5 in Appendix S3).

### 2.4 Species range: Rapoport's rule and climatic variability hypothesis

We explored the variations in species ranges with altitude using Stevens’ method (Stevens, 1989) and the cross‐species method (Letcher & Harvey, 1994). Stevens’ method compares the mean range size of the species co‐occurring in each 200 m band to the midpoint of the elevational band. The cross‐species method treats species as independent units and plots the range size against the midpoint of the species.

We tested the climatic variability hypothesis using the climate data from the WorldClim database ( http://www.worldclim.org) to understand the impact of candidate climate factors on the variation of range size.

### 2.5 Statistical analyses

We conducted simple ordinary least squares (OLS) and multiple regressions to evaluate the relationships between the SRP and each ecological and evolutionary predictor as well as the relationships between range size and each climate predictor. For the multiple regressions, we used the variance inflation factor (VIF) to assess the multicollinearity among predictors. After centering and standardization processing, the predictor variables with VIFs <3 were used for the multiple regressions. We also used a variance partitioning approach to estimate the relative importance of the variables on the SRP. All analyses were implemented in R packages “ggplot2” (Wickham, 2009) and “vegan” (Oksanen et al., 2013).

## 3 RESULTS

### 3.1 SRP

**Figure 2:**
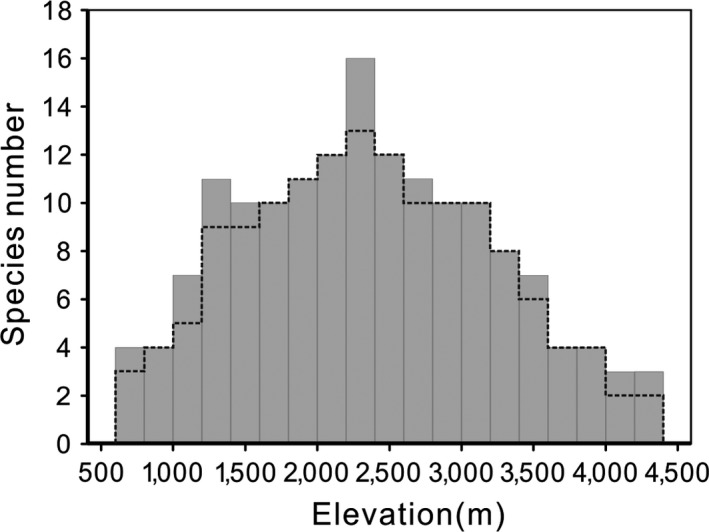
Species richness patterns along the elevational gradients from 600 to 4,300 m. The gray histogram is the species richness from the interpolation method; the broken line represents the observed records

The plateaus of the rarefaction curves indicated adequate sampling (see Fig. A9 in Appendix S3). The SRPs based on both interpolation and observed records were unimodal, peaking at the 2,200–2,400 m elevational band (Figure 2). The interpolation method considered both observed records and a vast amount of secondary data (see Appendix S2), which explicitly depicted the distribution boundary of the species. Thus, the interpolated SRP reflected a more reliable status of species richness and was used for all subsequent analyses in the study.

### 3.2 Ecological factors

**Figure 3:**
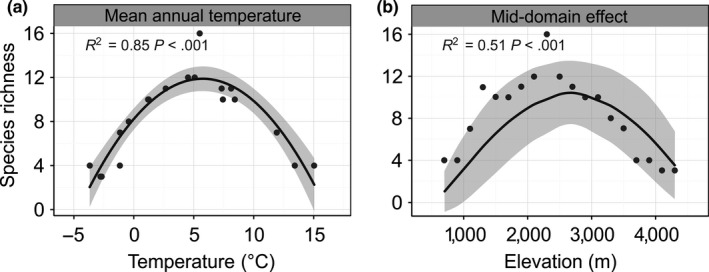
Relationships between the ecological factors and species richness. Only results with significant statistical relationships are displayed. (a) Relationship between mean annual temperature and species richness; the fitted black curve is from the OLS regression. (b) Relationship between the mid‐domain effect and species richness; the dots represent the species richness, the black curve is the predicted mean richness derived from RangeModel. Shaded areas show the 95% confidence interval of the fit or prediction

Among the series of climatic factors, only MAT showed a significant statistical relationship with the SRP (*R* ^2^ = 0.85, *p *<* *.001; Figure 3a; see also Fig. A10 in Appendix S3). For the spatial hypotheses, river basin area (*R* ^2^ = 0.01, *p *=* *.742) and total drainages area (*R* ^2^ = 0.00, *p *=* *.830) were not related to the SRP, but the MDE showed a strong correlation with the SRP (*R* ^2^ = 0.51, *p *<* *.001; Figure 3b; see also Fig. A11 in Appendix S3).

### 3.3 Evolutionary factors

A significant correlation was not observed between diversification rates and elevational gradients (see Fig. A12 in Appendix S3). The diversification rates of the stem group and the crown group were quite similar when three relative extinction rates (*e *=* *0, 0.45 and 0.9) were used. The results derived from BAMM also demonstrated nonsignificant variations of the diversification rates with altitude (see Fig. A13 in Appendix S3).

**Figure 4:**
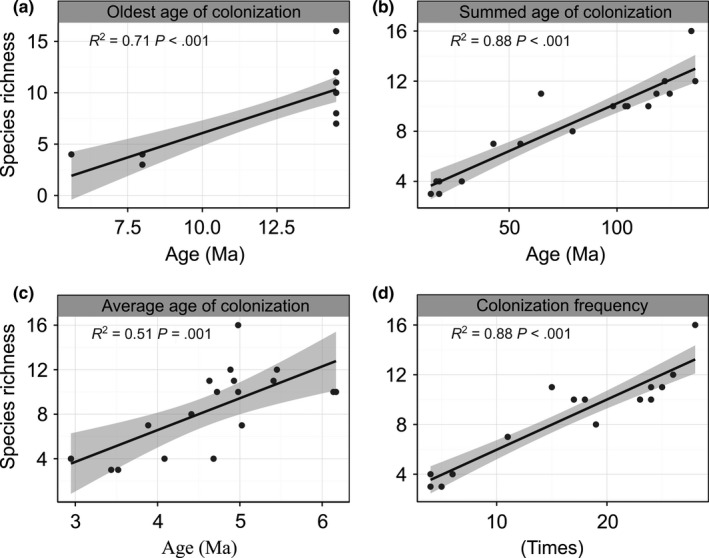
Relationships between species richness and (a) oldest age of colonization (b) summed age of colonization (c) average age of colonization and (d) colonization frequency. The fitted black lines are from the OLS regression. Shaded areas show the 95% confidence interval of the fit

The timing of colonization showed strong explanatory power for the SRP. The oldest age, summed age, and average age of colonization accounted for 71%, 88%, and 51% of the variation in the SRP, respectively. The colonization frequency also exhibited high statistical explanatory power for the SRP (*R* ^2^ = 0.88, *p *<* *.001; Figure 4).

### 3.4 Multivariate relationship of the SRP

**Figure 5:**
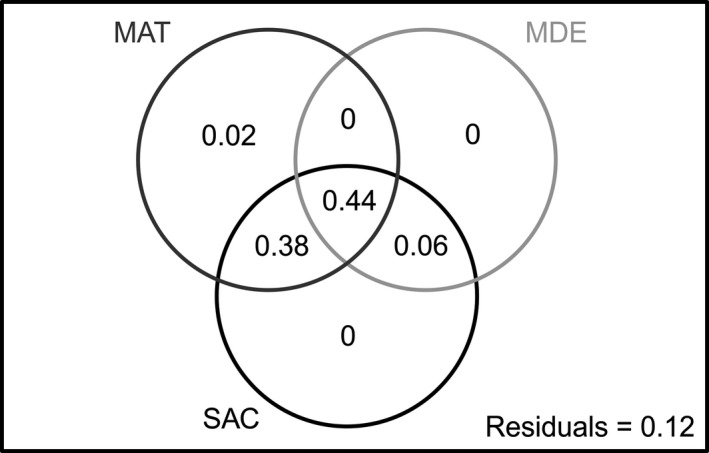
Result of the variance partitioning analysis showing the variation explained by three powerful variables (MAT, MDE, and SAC; Adjusted *R* ^2^). MAT, mean annual temperature; MDE, mid‐domain effect; SAC, summed age of colonization

We found that the partial regression coefficient of only the summed age of colonization (SAC) was significant in the multiple regression analysis (see Table A6 in Appendix S3). The variance partitioning result suggested that three powerful explanations (MAT, MDE, and SAC) had very high combined effects, but extremely low pure effects (Figure 5). The pure effect of MAT was 2%, and the other two effects (MDE and SAC) were 0%. The combined effect of the three explanations was 44%, while the combined effects of MAT‐SAC and MDE‐SAC were 38% and 6%, respectively.

### 3.5 Climate and species range size

**Figure 6:**
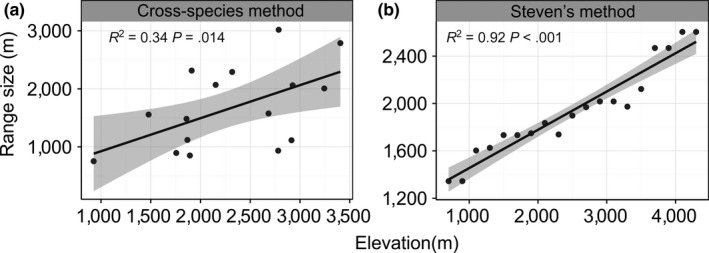
Test of Rapoport's rule for the ranges of Tibetan loaches along the elevational gradient. (a) cross‐species method (b) Steven's method

Both Stevens’ method and the cross‐species method revealed upward tendencies, which demonstrated that species at higher elevations tended to have wider ranges (Figure 6). The climatic factor results indicated that temperature and precipitation contributed to the variation of the range sizes with altitude (see Fig. A14 in Appendix S3). The MAT was negatively correlated with the range sizes at different altitudes (*R* ^2^ = 0.91, *p *<* *.001), while the AP exhibited a unimodal curve with range size (*R* ^2^ = 0.43, *p *=* *.011). PS (*R* ^2^ = 0.37, *p *=* *.006) and MDR (*R* ^2^ = 0.33, *p *=* *.010) were positively associated with range size, but TS (*R* ^2^ = 0.26, *p *=* *.089) and PDQ (*R* ^2^ = 0.00, *p *=* *.835) exhibited nonsignificant relations to the range size distribution. In total, 85% of the variation in range sizes was explained by a model that included MDR, PS, and PDQ (Table 1). The MDR and PS accounted for 55% of the variation in the range sizes.

**Table 1:** Summary of the multiple regression models of the effects of climatic factors (MDR, PS, and PDQ) and the climatic variability (MDR and PS) on the variation in range size

Source	Estimate	*SE*	*t*‐value	Significance	VIF
*Full model*					
Multiple R2 0.85; residual SE .425 on 15 df; p < .001					
MDR	.723	.117	6.167	<.001	1.367
PS	.769	.117	6.564	<.001	1.366
PDQ	.719	.131	5.503	<.001	1.702
*Reduced model*					
Multiple *R* ^2^ 0.55; residual *SE* .715 on 16 *df*;* p* = .002					
MDR	.435	.176	2.467	.025	1.095
PS	.482	.176	2.731	.015	1.095

*df*, degree of freedom; MDR, mean diurnal range; PDQ, precipitation of driest quarter; PS, precipitation seasonality; *SE*, standard errors; VIF, variance inflation factor.

## 4 DISCUSSION

### 4.1 SRPs

The SRP of Tibetan loaches on the northeastern margin of the Tibetan Plateau exhibited a unimodal pattern along the elevational gradients (Figure 2), which was consistent with most of the previous studies in the Tibetan Plateau and its marginal regions (e.g., plants, Wang, Tang, & Fang, 2007; fishes, Li et al., 2009; amphibians, Fu et al., 2006; reptiles, Fu et al., 2007; birds, Wu, Colwell, et al. 2013; mammals, Wu, Yang, et al. 2013). Hence, the unimodal shape is presumably the universal biota richness distribution pattern along the elevational gradients of the Tibetan Plateau and its adjacent highlands.

### 4.2 Ecological factors

Among the nine ecological factors examined, only MAT and MDE showed strong explanatory power for the current SRP of Tibetan loaches along elevational gradients (Figure 3).

The MAT was the only noteworthy climatic factor that shaped the elevational SRP of Tibetan loaches (Figure 3a; see also Fig. A10 in Appendix S3). It is widely believed that temperature influences species distribution via physiological constraints and food resource availability (Classen et al., 2015; Hawkins et al., 2003; McCain, 2007b). If the latter explanation is accepted, the richness of Tibetan loaches should be high at low elevations. Hence, the presumable interpretation of the outcome was that temperature had a strong relationship with the distribution of ectotherm loaches by affecting the physiological metabolism of the species.

The MDE prediction revealed that boundary constraints alone, without the influence of any other ecological factors, could also explain a significant proportion of the current SRP (Figure 3b). In the real‐world, physiology (temperature) and river basin limitations could serve as the boundary constraints. The species richness at midelevations surpassed the maximum value expected by the MDE and moved slightly toward low elevations, which indicated that the species richness at midelevations was driven by both ecological and evolutionary factors (see also Wu et al., 2014).

### 4.3 Evolutionary factors

Species richness in a geographic region is ultimately driven by speciation, extinction, and dispersal processes (Wiens & Donoghue, 2004). Unlike the ecological factors, the evolutionary factors predominately shaped the SRP over long time scales through the three processes above (Graham et al., 2014).

Our results indicated that the diversification rate was not the driver of the SRP (see Figs. A12 and A13 in Appendix S3), which is in accordance with many previous studies on other taxa (e.g., Wiens et al., 2007; Li et al., 2009; Wiens et al., 2009; Wu et al., 2014; but see Smith et al., 2007). This result is possibly attributed to the limited number of clades (Wu et al., 2014). However, the diversification rate hypotheses consider *in situ* species to some extent, which conflicts with the fact that the species distribution might shift along the elevational gradient over a long time scale (Svenning, Eiserhardt, Normand, Ordonez, & Sandel, 2015; Wu et al., 2014). The diversification rate hypotheses are more suited to describe species richness variations at greater scales (e.g., higher species richness in tropical than temperate zones, Mittelbach et al., 2007) and are less suited to describe variations over altitudinal gradients.

The timing of colonization and colonization frequency was supported as important factors in shaping the SRP (Figure 4). The timing of colonization responded to species accumulation. If the diversification rate had shifted much over time, the explanatory power of the colonization age would be questioned, particularly the oldest age of colonization (Wu et al., 2014). However, the changes in the diversification rate over evolutionary time would not disturb the explanatory power of the colonization frequency (Wu et al., 2014), which indicates dispersal events that could significantly change the species richness in a short time. Moreover, the SAC can be regarded as the colonization frequency with each event weighted by colonization age (Wu et al., 2014). Thus, the colonization frequency and the SAC were the better predictors for the SRP of Tibetan loaches. For the synthetical explanation and strong explanatory power, we employed the SAC as the evolutionary factor.

### 4.4 Multivariate relationship of the SRP

The three powerful explanations (MAT, MDE, and SAC) had very high combined effects but extremely low pure effects (Figure 5), indicating that the SRP was the result of the interaction of the three factors. The MDE assessed the geometric constraint. The MAT not only directly affected the distribution of the loaches but also could serve as a special environmental ‘geometric constraint.’ The SAC responded to species dispersal and accumulation throughout history, and this process was inevitably influenced by the two factors above.

### 4.5 Climatic variability hypothesis for species range

The present study revealed the tendency that the range of Tibetan loaches became larger with increasing altitude (Figure 6), corresponding well to Rapoport's elevational rule. The climatic variability factors (PS and MDR) provided a strong explanation for the variation in species ranges and exhibited positive relationships (Table 1; see also Fig. A14 in Appendix S3). The climatic variability hypothesis believes that species that can withstand broad climatic variability are able to become more widely distributed (Gaston & Chown, 1999). Our findings supported the climatic variability hypothesis as an explanation for the variation in the species ranges of the Tibetan loaches. While the precipitation indexes did not have relationships with the SRP, they were partly responsible for the variations in the species ranges with elevation. This result could be because the rainfall variation aggravated the instability of the hydrographic environment.

### 4.6 Rapoport's rule and SRP

Similar to previous studies, we found a combination of a peak the SRP at midelevations and Rapoport's elevational rule in the current work (Brehm et al., 2007; Fleishman et al., 1998; Sanders, 2002). Stevens (1992) invoked the climatic variability hypothesis (also cited as Rapoport “‘rescue”’ hypothesis) to explain the monotonic decrease in species richness with increasing elevation. However, it has been noted that the data provided by Stevens (1992) actually showed a peak at intermediate elevations (Colwell & Hurtt,^1994^; Rahbek,^1997^). Thus, this combination might be a common phenomenon in local species richness studies. Rather than a monotonic decrease, Rapoport's elevational rule is preferred to explain the peak in species richness at midelevations (Sanders, 2002). This combination was presumably the result of geometric constraints because climatic variability was conducive to larger species ranges and these larger ranges were more vulnerable to the MDE.

## 5 CONCLUSIONS

Tibetan loaches on the northeastern margin of the Tibetan Plateau displayed a peak in the SRP at midelevations. Our findings indicated that energy input, time‐for‐speciation, and species dispersal shaped the SRP directly or were mediated by geometric constraints. The current SRP was the outcome of complex interactions of biogeographical processes (e.g., species dispersal and accumulation) and regional ecological conditions (e.g., temperature and ecological constraints).

## CONFLICT OF INTEREST

None declared.

## AUTHOR CONTRIBUTIONS

C.F. and K.Z. conceived the ideas and led the writing. C.F. and Y.T. performed the analyses. Y.W. improved the ideas and analyses. Y.W., F.T., C.T., R.Z., and G.L. revised the manuscript. C.F., C.T., Y.T., R.Z., G.L., and K.Z. took part in the field works. K.Z. funded this work. All authors gave the final approval for publication.
