# Supplementary material for: Elevational diversity gradients of Tibetan loaches: The relative roles of ecological and evolutionary processes
Source: Ecol Evol. 2017 Oct 22;7(23):9970–7. doi: 10.1002/ece3.3504 (PMC5723583; doi:10.1002/ece3.3504)
Supplement: Supplementary file 1 [file ECE3-7-9970-s001.docx]

**Supplementary material for:**

*Elevational diversity gradients of Tibetan loaches: the relative roles of ecological and evolutionary processes*

**Appendix 1: Supplemental Materials and Methods**

**Field surveys and species richness**

Tibetan loaches (Cobitidae: *Triplophysa*) are important components of ichthyofauna of the Tibetan Plateau (Zhu, 1989; Wu & Wu, 1992; Chen et al., 1996). But the species identification among some species still keep inconsonant (He et al., 2011). To ascertain the species composition of study area, we carried out repeated field surveys covering the entire region from 2012 to 2015. All the surveys were conducted under the permission of fisheries departments of Chinese government. Species identification followed morphology as well as genetic markers based on extensive samples. Specimens used in this study were deposited in the collection of the Northwest Institute of Plateau Biology, Chinese Academy of Sciences, Xining. After diagnosis, total nineteen valid Tibetan loach species were detected in this region, including two undescribed species. To assess the adequacy of sampling, we used EstimateS ver. 8.2 (Colwell, 2009) to compute the expected number of species (Fig. A9 in Appendix 3). Plateau of the species accumulation curves assume adequate surveys of species (Wu et al., 2013).

Observed species richness is the total number of species detected in each elevational band in our field surveys (Walther & Moore, 2005). To ascertain species ranges as accurately as possible, we also consult intensive secondary data (Appendix 2). Assuming species occur at whole elevation between the threshold values, species richness based on such rang sizes is considered as interpolated richness (Colwell et al., 2004; McCain, 2004; Li et al., 2009). Although the authenticity of mid-altitude bulge derived from interpolation always is doubted (Grytnes & Vetaas, 2002; Wu et al., 2013), the interpolation is often considered a valid approach for vagile species along local gradients where suitable habitat is continuous (Wu et al., 2013). Species richness based on observed records displays a unimodal shape along altitude and with a peak at 2300 m band, which gives nearly uniform pattern with that based on interpolation (*R*^2^=0.94). Thus, interpolated richness is expected to reflect the more reliable status of species richness and used for all the subsequent analyses in this study.

**Phylogenetic hypothesis of Tibetan loaches**

To reconstruct the phylogenetic relationships of Tibetan loaches, we amplified the complete mitochondrial cytochrome *b* gene (1140 bp), which is extensively utilized for molecular phylogenetic evaluations by reason of its good performance in phylogenetic reconstruction in species- and genus- level (Zardoya & Meyer 1996; He et al., 2006; Kartavtsev et al., 2007). Primers used for PCR followed Xiao et al. (2001). Following previous phylogenetic study of Tibetan loaches (He et al., 2006), we chose *Barbatula nuda* and *Schistura balteata* as outgroup species. In order to date the divergence among taxa, we also add *Triplophysa strauchii* into the phylogenetic analyses. All the sequences were deposited in GenBank and accession numbers were listed in the Table A2 in Appendix 3.

DNA sequences were aligned using CLUSTALW (Thompson et al., 1994), which is integrated with MEGA 6.0 (Tamura et al., 2013). The phylogenetic analyses were performed using maximum likelihood (ML) approach in Phyml 3.0 (Guindon et al., 2010) and the Bayesian inference (BI) of likelihood in MRBAYES 3.2.0 (Ronquist et al., 2012). The best model of DNA substitution (HKY+I+G) identified by jModelTest 0.1.1 (Guindon & Gascuel 2003; Posada, 2008) based on Bayesian Information Criterion (BIC) (Schwarz, 1978; Luo et al., 2010) was implemented in the ML and BI analyses. To assess statistical support for hypothesized clades, bootstrap analysis was performed with 1000 replicates for the ML tree. For BI, the posterior distributions were obtained by Markov Chain Monte Carlo (MCMC) analysis with one cold chain and three heated chains. Samples of trees and the parameters were drawn every 100 steps from a total of 2,000,000 MCMC generations, with the first 25% samples discarded as burn-in.

The two undescribed species distributed in the edge of the study area and with few sample sites, which may cause undervaluation of species ranges. To minimize the deviation of range estimation in MESQUITE, both species were removed. Removing the two species made no difference on the estimation of diversification rates, and yielded unanimous topological trees for the remaining species (Figs. A4 and A5 in Appendix 3).

We took advantage of unique geological events (Gonghe Movement and Tianshan Mountains uplift) to estimate the divergent time due to the absence of fossil records. Gonghe Movement (*c.* 0.15 Ma) triggered the upheaval of the Riyue mountain, which separated the Lake Qinghai from the Yellow River (Li et al., 2001). The Tianshan Mountains uplift (*c.* 2.58 - 7 Ma) broke the contact between Ili River system and Junggar river system (Sun et al., 2004). Therefore, we assumed geographic isolation induced by these two geological events promoted the genetic differentiation between Lake Qinghai and the Yellow River populations of *Triplophysa scleroptera* as well as between the Ili River system and the Junggar River populations of *Triplophysa strauchii*. Based on this assumption, we used the timing of the two geological events as time calibrations to infer the divergence among taxa. But the timing of Tianshan Mountains uplift (*c.* 2.58 - 7 Ma) is a wide range of time. Recent study implemented by Li et al. (2015) on *Gymnodiptychus dybowskii* has pointed out that the time of divergence was 2.92 Mya (95% HPD = 2.40 - 3.44) between the Ili River and Junggar Basin clades, located in the timing of orogeny. Thus, we used 0.15 Ma and 2.92 Ma as time calibrations for the last common ancestor of separate populations of *Triplophysa scleroptera* and *Triplophysa strauchii*, respectively.

The divergence time was estimated using a Lognormal relaxed clock model in BEAST v1.8.0 (Drummond et al., 2012), with the HKY+I+G substitution model and the Yule prior approach. The analysis sampled every 1000 generations for 20 million generations with 20% of samples discarded as burn-in. The effective sample size (ESS) for parameter estimates and convergence was checked using TRACER 1.5 (Rambaut & Drummond 2007) to insure all the ESSs reaching at least 200. This estimation was conducted twice using the same parameter settings to generate robust results. Only the first outcomes were used.

The results indicated that the last common ancestor of *Triplophysa robusta* and *Triplophysa hsutschouensis*, which mirrored the connection of the Yellow River and the Hexi rivers system, occurred at *c.* 1.65 Ma B. P. (Fig. A6 in Appendix 3). Previous studies (Li et al., 1996; Li et al., 2001) on the Yellow River terrace in Lanzhou basin and eastern Qilian Mountains uncovered that the oldest terrace in north shore Yellow River (the seventh terrace) was dated to 1.6 Ma B. P., during this period contemporary drainage pattern of Yellow River had come into being, which indicated intense tectonic movement. Besides, the time estimation was also in accordance with the results of Zhao (2009) in Schizothoracine fish (1.50 – 2.44 Ma). Furthermore, ancestral trait reconstruction simulated the last common ancestor of all Tibetan loaches (except *Triplophysa yarkandensis*, which clustered with other genera) had been at elevational midpoint 2365 m (elevational range 1368.8 – 3361.2 m) during the period of 14.4 – 19.2 Ma B. P.. This may suggest that the Tibetan Plateau in this region had reached *c.* 2365 m in height in this period, corresponding to the studies on paleogeology and paleoclimate (Shi et al., 1999; Li et al., 2001; Zhang et al., 2006). Hence, we accepted the accuracy of the time estimation.

**References**

Chen, Y., Chen, Y. & Liu, H. (1996). Studies on the position of the Qinghai-Xizang Plateau region in zoogeographic divisions and its eastern demarcation line. *Acta Hydrobiologica Sinica*, **20**, 97-103.

Colwell, R.K., Rahbek, C. & Gotelli, N.J. (2004). The mid-domain effect and species richness patterns: what have we learned so far? *The American Naturalist*, **163**, E1-E23.

Colwell, R.K. (2009). EstimateS and User’s Guide: Statistical estimation of species richness and shared species from samples. Ver. 8.2. Persistent URL <http://purl.oclc.org/estimates/>.

Drummond, A.J., Suchard, M.A., Xie, D. & Rambaut, A. (2012). Bayesian phylogenetics with BEAUti and the BEAST 1.7. *Molecular Biology and Evolution*, **29**, 1969-1973.

Grytnes, J.A. & Vetaas, O.R. (2002). Species richness and altitude: a comparison between null models and interpolated plant species richness along the Himalayan altitudinal gradient, Nepal. *The American Naturalist*, **159**, 294-304.

Guindon, S. & Gascuel, O. (2003). A simple, fast, and accurate algorithm to estimate large phylogenies by maximum likelihood. *Systematic Biology*, **52**, 696-704.

Guindon, S., Dufayard, J.-F., Lefort, V., Anisimova, M., Hordijk, W. & Gascuel, O. (2010). New algorithms and methods to estimate maximum-likelihood phylogenies: assessing the performance of PhyML 3.0. *Systematic Biology*, **59**, 307-321.

He, C., Song, Z. & Zhang, E. (2011). *Triplophysa* fishes in China and the status of its taxonomic studies. *Sichuan Journal of Zoology*, **30**, 150-155.

He, D., Chen, Y. & Chen, Y. (2006). The molecular phylogeny and biogeography of genus *Triplophysa*. *Progress in Natural Science*, **16**, 1395-1404.

Kartavtsev, Y., Park, T.-J., Vinnikov, K., Ivankov, V., Sharina, S. & Lee, J.-S. (2007). Cytochrome b (Cyt-b) gene sequence analysis in six flatfish species (Teleostei, Pleuronectidae), with phylogenetic and taxonomic insights. *Marine Biology*, **152**, 757-773.

Li, G., Peng, Z., Zhang, R., Tang, Y., Tong, C., Feng, C., Zhang, C. & Zhao, K. (2015). Mito-nuclear phylogeography of the cyprinid fish *Gymnodiptychus dybowskii* in the arid Tien Shan region of Central Asia. *Biological Journal of the Linnean Society*, **118**, 304-314.

Li, J., Fang, X., Pan, B., Zhao, Z. & Song, Y. (2001). Late Cenozoic intensive uplift of Qinghai-Xizang Plateau and its impacts on environments in surrounding area. *Quaternary Sciences*, **21**, 381-391.

Li, J., Fang, X., Ma, H., Zhu, J., Pan, B. & Chen, H. (1996). Geomorphic evolution in upper reaches of Yellow River and the uplift of Qinghai-Xizang Plateau in late Cenozoic. *Science in China*, **26**, 316-322.

Li, J., He, Q., Hua, X., Zhou, J., Xu, H., Chen, J. & Fu, C. (2009). Climate and history explain the species richness peak at mid-elevation for Schizothorax fishes (Cypriniformes: Cyprinidae) distributed in the Tibetan Plateau and its adjacent regions. *Global Ecology and Biogeography*, **18**, 264-272.

Luo, A., Qiao, H., Zhang, Y., Shi, W., Ho, S.Y., Xu, W., Zhang, A. & Zhu, C. (2010). Performance of criteria for selecting evolutionary models in phylogenetics: a comprehensive study based on simulated datasets. *BMC Evolutionary Biology*, **10**, 1.

McCain, C.M. (2004). The mid-domain effect applied to elevational gradients: species richness of small mammals in Costa Rica. *Journal of Biogeography*, **31**, 19-31.

Posada, D. (2008). jModelTest: phylogenetic model averaging. *Molecular Biology and Evolution*, **25**, 1253-1256.

Rambaut, A. & Drummond, A. (2007). TRACER version 1.5. Available: http. *beast. bio. ed. ac. uk/Tracer*.

Ronquist, F., Teslenko, M., van der Mark, P., Ayres, D.L., Darling, A., Höhna, S., Larget, B., Liu, L., Suchard, M.A. & Huelsenbeck, J.P. (2012). MrBayes 3.2: efficient Bayesian phylogenetic inference and model choice across a large model space. *Systematic Biology*, **61**, 539-542.

Schwarz, G. (1978). Estimating the dimension of a model. *The Annals of Statistics*, **6**, 461-464.

Shi, Y., Li, J., Li, B., Yao, T., Wang, S., Li, S., Cui, Z., Wang, F., Pan, B., Fang, X. & Zhang, Q. (1999). Uplift of the Qinghai-Xizang (Tibetan) plateau and east Asia environmental change during late Cenozoic. *Acta Geographica Sinica*, **54**, 11-20.

Sun, J., Zhu, R. & Bowler, J. (2004). Timing of the Tianshan Mountains uplift constrained by magnetostratigraphic analysis of molasse deposits. *Earth and Planetary Science Letters*, **219**, 239-253.

Tamura, K., Stecher, G., Peterson, D., Filipski, A. & Kumar, S. (2013). MEGA6: molecular evolutionary genetics analysis version 6.0. *Molecular Biology and Evolution*, mst197.

Thompson, J.D., Higgins, D.G. & Gibson, T.J. (1994). CLUSTAL W: improving the sensitivity of progressive multiple sequence alignment through sequence weighting, position-specific gap penalties and weight matrix choice. *Nucleic Acids Research*, **22**, 4673-4680.

Walther, B.A. & Moore, J.L. (2005). The concepts of bias, precision and accuracy, and their use in testing the performance of species richness estimators, with a literature review of estimator performance. *Ecography*, **28**, 815-829.

Wu, Y. & Wu, C. (1992). *The fishes of the Qinghai-Xizang plateau*. Sichuan Publishing House of Science & Technology.

Wu, Y., Yang, Q., Wen, Z., Xia, L., Zhang, Q. & Zhou, H. (2013). What drives the species richness patterns of non‐volant small mammals along a subtropical elevational gradient? *Ecography*, **36**, 185-196.

Xiao, W., Zhang, Y. & Liu, H. (2001). Molecular systematics of Xenocyprinae (Teleostei: Cyprinidae): taxonomy, biogeography, and coevolution of a special group restricted in East Asia. *Molecular Phylogenetics and Evolution*, **18**, 163-173.

Zardoya, R. & Meyer, A. (1996). Phylogenetic performance of mitochondrial protein-coding genes in resolving relationships among vertebrates. *Molecular Biology and Evolution*, **13**, 933-942.

Zhang, P.-Z., Zheng, D.-W., Yin, G.-M., Yuan, D.-Y., Zhang, G.-L., Li, C.-Y. & Wang, Z.-C. (2006). Discussion on late Cenozoic growth and rise of northeastern margin of the Tibetan Plateau. *Quaternary Sciences*, **26**, 5-13.

Zhao, K. (2009). *Adaptive evolution and biogeography in the Schizothoracine fish (Cyprinidae) endemic to the northeast Tibetan Plateau*. Northweat Institute of Plateau Biology, Chinese Academy of Sciences, Xingning.

Zhu, S. (1989). *The loaches of the subfamily Nemacheilinae in China (Cypriniformes: Cobitidae)*. Jiangsu Science and Technology Publishing House.
