# Supplementary material for: Elevational diversity gradients of Tibetan loaches: The relative roles of ecological and evolutionary processes
Source: Ecol Evol. 2017 Oct 22;7(23):9970–7. doi: 10.1002/ece3.3504 (PMC5723583; doi:10.1002/ece3.3504)
Supplement: Supplementary file 2 [file ECE3-7-9970-s002.docx]

**Supplementary material for:**

*Elevational diversity gradients of Tibetan loaches: the relative role of ecological and evolutionary processes*

**Appendix 2: Literature sources for information of distribution and altitude of Tibetan loaches**

Cao, W. & Wu, X. (1962) Ichthyology and fishery problems in Ganzi and Aba region in western of Sichuan Province. *Acta Hydrobiologica Sinica*, **2**, 79-110.

Chen, J., Xu, T., Fang, S., Song, S. & Wang, X. (1987) *Fishes in Qingling Mountain area*. Beijing: Science Press.

Ding, R. (1994) The fishes of Sichuan. *Chengdu, Sichuan, China: Sichuan Publishing House of Science and Technology*,

Ding, R. (1996) A research for fauna and protection of fishes in Yellow river system in Sichuan Province. *Chinese Journal of Zoology*, **31**, 8-11.

Ding, R., Fang, S. & Fang, J. (1996) Studies on the DNA fingerprinting in two species of the genus Triplophysafrom China with description of a new species (Cyprinifomes: Cobitidae). *Sichuan Journal of Zoology*, **15**, 10-15.

Fang, S., Xu, T., Song, S., Wang, X. & Chen, J. (1984) A study on fauna of the fishes in Shanxi Province, China. *Journal of Lanzhou University* (*Natural Sciences*), **20**, 97-115.

Guo, Y., Zhang, R. & Cai, L. (2012) *Fishes of Xinjiang Uygur Autonomous Region, China*. Xin Jiang Science and Technology Press, Urumqi.

He, D., Chen, Y. & Chen, Y. (2006) The molecular phylogeny and biogeography of genus Triplophysa. *Progress in Natural Science*, **16**, 1395-1404.

He, R. (1982) A research of fish fauna and fishery on Liujiaxia to Shizuishan stretches in the Yellow River. *Ningxia Journal of Agriculture*, **1**, 35-37.

Li, S. & Chang, S. (1974) Two new species and one new subspecies of fishes from the northern part of Kansu Province, China. *Acta Zoologica Sinica*, **20**, 414-419.

Liu, W.-j., Huang, Z.-h. & Liu, N.-f. (2003) Studies on the ichthyofauna of Maiji Mountain area in Gansu province. *Journal of Lanzhou University* (*Natural Sciences*), **39**, 68-70.

Liu, Y. (1981) Fishery situation and discussion on the ways of proliferation in Maqu section of Yellow River. *Freshwater Fisheries*, **3**, 37-39.

Ma, G. & Zhang, M. (2007) A study of the species diversity of fish resource in Tianshui. *Journal of Tianshui Normal University*, **27**, 29-31.

Ma, G. & Zhang, M. (2014) A research of fish resources diversity in southeast Gansu. *Journal of Tianshui Normal University*, **34**, 21-25.

Qu, X. (2009) *Study on aquatic germplasm resources protection district of Maqu waters of the Yellow River*. Graduate School of Chinese Academy of Agricultural Sciences, Beijing.

Shi, B. & Deng, Q. (1980) Checklist and brief survey history of fishes in Jialing River. *Journal of Southwest Normal University*, **2**, 34-44.

Song, S. & Wang, X. (1983) Notes on fishes fauna of the upper reaches of Weihe River in China. *Journal of Lanzhou University* (*Natural Sciences*), **19**, 120-128.

Tang, W. & He, D. (2013) Fish resource survey on Cihaxia to Jishixia stretches in the upper Yellow River (2005–2010). *Journal of Lake Sciences*, **25**, 600-608.

Tang, W., Chen, Y. & Ding, C. (2013) The current situation and protection of fish resources in Huangshui River in Qinghai province. *Journal of Dalian Ocean University*, **28**, 307-313.

Wang, T., Zhang, Y., Guan, L., Du, Y., Lou, Z. & Jiao, W. (2015) Current freshwater fish resources and the application of DNA barcoding in species identification in Gansu Province. *Biodiversity Science*, **23**, 306-313.

Wang, X. (1991) Vertebrate fauna of Gansu. *Lanzhou: Gansu Science and Technology Publishing House*, **1**, 1.

Wang, X. & Zhu, S. (1979) On a new species of the genus *Nemachilus* in Gansu Province, China. *Journal of Lanzhou University* (*Natural Sciences*), **4**, 129-132.

Wang, X., Qin, C., Cui, W. & Fan, Y. (1974) Survey of fish resources in Bailong River and several advises for utilize. *Chinese Journal of Zoology*, **1**, 3-8.

Wu, W., Xu, Z., Yin, X. & Yu, Y. (2014) Fish community structure and biological integrity in the Wei River basin. *Research of Environmental Sciences*, **27**, 981-989.

Wu, Y. & Chen, Y. (1979) Notes on fishes from Golog and Yushu region of Qinghai Province, China. *Acta Zootaxonomica Sinica*, **4**, 287-296.

Wu, Y. & Wu, C. (1984) Notes on fishes from lake Summ Cuo of Qinghai Province, China. *Acta Zootaxonomica Sinica*, **9**, 326-329.

Wu, Y. & Wu, C. (1992) *The fishes of the Qinghai-Xizang plateau*. Sichuan Publishing House of Science & Technology.

Wu, Y., Yu, D., Wu, C., Jing, C. & Chen, Y. (1994) A preliminary study on the resources of fishes and conservation in Hohxil (Kokoxili) region of Qinghai Province. *Chinese Journal of Zoology*, **29**, 9-17.

Xu, T. & Li, Z. (1984) Studies on fishes fauna of the Weihe River. *Journal of Xinxiang Normal College*, **4**, 73-78.

Yang, G. & Xie, C. (1983) A new species of Cobitid fishes from upper Changjiang River. *Acta Zootaxonomica Sinica*, **8**, 314-316.

Yang, Y. & Zhang, Y. (1991) Study on fishes' fauna and evolution of the inland river, Hexi corridor. *Journal of Lanzhou University* (*Natural Sciences*), **27**, 141-144.

Yang, Y. & Tang, Y. (1995) Resources and geographical distribution of the fishes in Gansu Province, China. *Journal of Gansu Sciences*, **3**, 03.

Zhang, C. & Zhang, Y. (1965) Several fishes in lake Zhaling and Datong river of Qinghai province. *Chinese Journal of Zoology*, **3**, 121-122.

Zhang, D., Zhang, X., Dai, Z., Dei, P. & Lu, J. (1998) Morphological change of Triplophysa siluroides in Ningxia. *Journal of Ningxia Agricultural College*, **19**, 24-26.

Zhang, F. (2013) *Phylogeography of the Triplophysa leptosome in Qilian mountains*. Lanzhou University, Lanzhou.

Zhang, H., Zhong, L., Yang, J., Mao, Z., Yang, G., Li, Y. & Xu, T. (2006) On fish species diversity of Hanjiang River in Shaanxi province. *Journal of Shaanxi Normal University* (*Natural Science Edition*), **34**, 60-66.

Zhang, Y., Lou, Z., Wang, T., Du, Y. & Jiao, W. (2013) Population genetic diversity of *Triplophysa siluroides* in Maqu section in upper reaches of Yellow River. *Freshwater Fisheries*, **43**, 86-89.

Zhao, T. (1982) Fishes of Abogain Gol River in Nei Mongol Ziziqu, China. *Journal of Lanzhou University* (*Natural Sciences*), **18**, 112-118.

Zhao, T. (1984) Taxonomic Problems of Some Nemachilus Fishes. *Zoological Research*, **5**, 341-346.

Zhao, T. (1991) Fish-fauna and zoogeographical division of Hexi-Alashan region, the northwest china. *Acta Zoologica Sinica*, **37**, 153-167.

Zhao, T. & Wang, X. (1988) Fishes and fishery problems of Hexi Area, Gansu Province, with a descriptions of a new subspecies and an unidentified species of Cobitidae. *Journal of Lanzhou University* (*Natural Sciences*), **24**, 109-119.

Zhu, S. (1989) *The loaches of the subfamily Nemacheilinae in China (Cypriniformes: Cobitidae)*. Jiangsu Science and Technology Publishing House.

Zhu, S. & Wu, Y. (1975) The fish fauna study in Qinghaihu lake. *The Fish Fauna of Qinghai Lake Region and biology of Gymnocypris przewalskii przewalskii (Kessler)* (ed. by Q.P. Institute of Biology). Science Press, Beijing.

Zhu, S. & Wu, Y. (1981) A new species and a new subspecies of loaches of the genus *Nemachilus* from Qinghai Province. *Acta Zootaxonomica Sinica*, **6**, 221-224.
