# Supplementary material for: Elevational diversity gradients of Tibetan loaches: The relative roles of ecological and evolutionary processes
Source: Ecol Evol. 2017 Oct 22;7(23):9970–7. doi: 10.1002/ece3.3504 (PMC5723583; doi:10.1002/ece3.3504)
Supplement: Supplementary file 3 [file ECE3-7-9970-s003.docx]

**Supplementary material for:**

*Elevational diversity gradients of Tibetan loaches: the relative roles of ecological and evolutionary processes*

**Appendix 3: Supplemental Tables and Figures**

**Table A1** Species checklist of the Tibetan loaches and the information of distribution and altitude. Asterisk denotes the poorish sampling sites.

| Species | Altitude upper limit | Altitude lower limit | Range size | Altitude midpoint |
| --- | --- | --- | --- | --- |
| *Triplophysa hsutschouensis* | 3464 | 1173 | 2291 | 2318.5 |
| *Triplophysa stoliczkae* | 3187 | 1118 | 2069 | 2152.5 |
| *Triplophysa wuweiensis* | 2319 | 1468 | 851 | 1893.5 |
| *Triplophysa tenuis* | 2428 | 1309 | 1119 | 1868.5 |
| *Triplophysa yarkandensis* | 2204 | 1309 | 895 | 1756.5 |
| *Triplophysa leptosoma* | 4800 | 2012 | 2788 | 3406 |
| *Triplophysa pseudoscleroptera* | 3472 | 1896 | 1576 | 2684 |
| *Triplophysa minxianensis* | 2601 | 1118 | 1483 | 1859.5 |
| *Triplophysa dalaica* | 2257 | 700 | 1557 | 1478.5 |
| *Triplophysa robusta* | 3069 | 754 | 2315 | 1911.5 |
| *Triplophysa pappenheimi* | 3957 | 1896 | 2061 | 2926.5 |
| *Triplophysa siluroides* | 4295 | 1277 | 3018 | 2786 |
| *Triplophysa scleroptera* | 4248 | 2241 | 2007 | 3244.5 |
| *Triplophysa orientalis* | 3472 | 2358 | 1114 | 2915 |
| *Triplophysa alticeps* | 3248 | 2314 | 934 | 2781 |
| *Triplophysa bleekeri* | 1303 | 550 | 753 | 926.5 |
| *Triplophysa polyfasciata* | 1303 | 550 | 753 | 926.5 |
| *Triplophysa* sp1* |  |  |  | 2380 |
| *Triplophysa* sp2* | 1768 | 1360 | 408 | 1564 |

**Table A2** GenBank accession numbers and the location of the examined species

| Species | Accession number | Origin |
| --- | --- | --- |
| *Triplophysa hsutschouensis* | KX373852 | Beidahe river, Sunan, Gansu |
| *Triplophysa stoliczkae* | KX373851 | Daxiahe river, Linxia, Gansu |
| *Triplophysa wuweiensis* | KX373838 | Shiyanghe river, Wuwei, Gansu |
| *Triplophysa tenuis* | KX373841 | Heihe (Hexi drainage), Zhangye, Gansu |
| *Triplophysa yarkandensis* | KX373836 | Danghe river, Dunhuang, Gansu |
| *Triplophysa leptosoma* | KX373839 | Datonghe river, Menyuan, Qinghai |
| *Triplophysa pseudoscleroptera* | KX373844 | Taohe river, Lintao, Gansu |
| *Triplophysa minxianensis* | KX373849 | Weihe river, Qingshui, Gansu |
| *Triplophysa dalaica* | KX373845 | Weihe river, Wushan, Gansu |
| *Triplophysa robusta* | KX373850 | Daxiahe river, Linxia, Gansu |
| *Triplophysa pappenheimi* | KX373843 | Taohe river, Luqu, Gansu |
| *Triplophysa siluroides* | KX373842 | Yellow river, Maduo, Qinghai |
| *Triplophysa scleroptera* | KX373840 | Yellow river, Guide, Qinghai |
| *Triplophysa scleroptera* 1 | KX373833 | Lake Qinghai, Qinghai |
| *Triplophysa orientalis* | KX373846 | Heihe (Yellow river), ruoergai, Sichuan |
| *Triplophysa alticeps* | KX373837 | Lake Qinghai, Qinghai |
| *Triplophysa bleekeri* | KX373847 | Baishui river, Wenxian, Gansu |
| *Triplophysa polyfasciata* | KX373848 | Baishui river, Wenxian, Gansu |
| *Triplophysa* sp1 | KX373835 | Heihe (Yellow river), ruoergai, Sichuan |
| *Triplophysa* sp2 | KX373834 | Weihe river, Qingshui, Gansu |
| *Triplophysa strauchii* 1 | KX373853 | Ili river, Huocheng, Xinjiang |
| *Triplophysa strauchii* 2 | KX373854 | Junggar drainage, Qitai, Xinjiang |
| *Barbatula nuda* | NC_022858 | Zhao *et al.*, 2015 |
| *Schistura balteata* | AB242172 | Saitoh *et al.*, 2006 |

**Table A3** Estimation of diversification rates of Tibetan loaches using the method-of-moments estimator (Magallon & Sanderson, 2001). The diversification rates are estimated using stem and crown group age based on three particular values of relative extinction rates (E=0, 0.45, 0.9). Predicted elevation is the elevational midpoint of last common ancestor of each clade predicted by MESQUITE. Average elevation is the average elevational midpoint of total species in a clade.

| **Clade** | **richness** | **Diversification rate** | | | | | | **Elevation (m)** | |
| --- | --- | --- | --- | --- | --- | --- | --- | --- | --- |
|  |  | **Stem group age** | | | **Crowm group age** | | | **Predicted** | **Average** |
|  |  | **E=0** | **E=0.45** | **E=0.9** | **E=0** | **E=0.45** | **E=0.9** |  |  |
| **1** | 5 | 0.1111 | 0.0803 | 0.0232 | 0.1632 | 0.1448 | 0.0497 | 2392.8 | 2360.4 |
| **2** | 11 | 0.2254 | 0.1760 | 0.0652 | 0.2553 | 0.2305 | 0.0958 | 2282.1 | 2245.1 |
| **3** | 3 | 0.1033 | 0.0697 | 0.0171 | 0.0507 | 0.0456 | 0.0147 | 2399.9 | 1753 |
| **4** | 2 | 0.1234 | 0.0780 | 0.0170 | 0 | 0 | 0 | 2786 | 2856.3 |
| **5** | 3 | 0.1956 | 0.1321 | 0.0325 | 0.1080 | 0.0971 | 0.0314 | 2182.8 | 2029.8 |
| **6** | 9 | 0.3914 | 0.3004 | 0.1047 | 0.3213 | 0.2885 | 0.1139 | 2525.3 | 2404.9 |
| **7** | 1 | 0 | 0 | 0 | - | - | - | 1478.5 | 1478.5 |
| **8** | 1 | 0 | 0 | 0 | - | - | - | 1893.5 | 1893.5 |
| **9** | 2 | 0.0867 | 0.0548 | 0.0119 | 0 | 0 | 0 | 1454.9 | 926.5 |
| **10** | 1 | 0 | 0 | 0 | - | - | - | 3406 | 3406 |
| **11** | 10 | 0.3449 | 0.2671 | 0.0961 | 0.2867 | 0.2581 | 0.1047 | 2366.4 | 2289.1 |

The list of species in clade 1-11 is marked in Fig. A6.

**Table A4** Diversification rates of each clade estimated by BAMM model (Rabosky et al., 2014).

| **Clade** | **richness** | **Diversification rate** | | |
| --- | --- | --- | --- | --- |
|  |  | **Mean** | **Median** | **95%CI** |
| **1** | 5 | 0.1431 | 0.1549 | (-0.0330, 0.2922) |
| **2** | 11 | 0.1758 | 0.1789 | (0.0195, 0.3184) |
| **3** | 3 | 0.1390 | 0.1505 | (-0.0507, 0.2874) |
| **4** | 2 | 0.1636 | 0.1724 | (-0.0154, 0.3131) |
| **5** | 3 | 0.1442 | 0.1664 | (-0.1041, 0.3115) |
| **6** | 9 | 0.1799 | 0.1819 | (-0.0149, 0.3297) |
| **7** | 1 | 0.1677 | 0.1796 | (-0.0285, 0.3233) |
| **8** | 1 | 0.1646 | 0.1778 | (-0.0595, 0.3261) |
| **9** | 2 | 0.1799 | 0.1541 | (-0.1423, 0.2941) |
| **10** | 1 | 0.1414 | 0.1724 | (-0.0663, 0.3138) |
| **11** | 10 | 0.1793 | 0.1820 | (0.0132, 0.3299) |

The list of species in clade 1-11 is marked in Fig. A6.

**Table A5** Timing of colonization and colonization frequency of Tibetan loaches in the 19 elevational bands.

| **Elevational band (m)** | **Species richness** | **Colonization age (Ma)** | | | **Colonization frequency** |
| --- | --- | --- | --- | --- | --- |
|  |  | **Oldest age** | **Summed age** | **Average age** |  |
| **600-800** | 4 | 5.614 | 16.336 | 4.084 | 4 |
| **800-1000** | 4 | 7.992 | 28.081 | 4.680 | 6 |
| **1000-1200** | 7 | 14.484 | 55.262 | 5.024 | 11 |
| **1200-1400** | 11 | 14.484 | 64.816 | 4.630 | 15 |
| **1400-1600** | 10 | 14.484 | 98.183 | 6.136 | 17 |
| **1600-1800** | 10 | 14.484 | 104.860 | 6.168 | 18 |
| **1800-2000** | 11 | 14.484 | 124.406 | 5.409 | 24 |
| **2000-2200** | 12 | 14.484 | 136.256 | 5.450 | 26 |
| **2200-2400** | 16 | 14.484 | 134.428 | 4.979 | 28 |
| **2400-2600** | 12 | 14.484 | 122.137 | 4.885 | 26 |
| **2600-2800** | 11 | 14.484 | 118.280 | 4.928 | 25 |
| **2800-3000** | 10 | 14.484 | 114.526 | 4.979 | 24 |
| **3000-3200** | 10 | 14.484 | 103.888 | 4.722 | 23 |
| **3200-3400** | 8 | 14.484 | 79.401 | 4.411 | 19 |
| **3400-3600** | 7 | 14.484 | 42.744 | 3.886 | 11 |
| **3600-3800** | 4 | 7.992 | 17.683 | 2.947 | 6 |
| **3800-4000** | 4 | 7.992 | 17.683 | 2.947 | 6 |
| **4000-4200** | 3 | 7.992 | 17.603 | 3.521 | 5 |
| **4200-4400** | 3 | 7.992 | 13.745 | 3.436 | 4 |

**Table A6** Summary of the multiple regression model for the effects of ecological factors (MDE, AP and Area) and evolutionary factor (SAC) on species richness (SRP).

| **Source** | **Estimate** | **SE** | ***t*-value** | **Significance** | **VIF** |
| --- | --- | --- | --- | --- | --- |
| *Multiple R^2^ 0.89*; *residual SE 0.38 on 14 df*; *P<0.001* | | | | | |
| MDE | -0.086 | 0.155 | -0.555 | 0.588 | 2.958 |
| **SAC** | 1.001 | 0.150 | 6.698 | **<0.001** | 2.763 |
| AP | 0.023 | 0.129 | -0.179 | 0.861 | 2.050 |
| Area | 0.069 | 0.131 | 0.531 | 0.604 | 2.113 |

AP, annual precipitation; df, degree of freedom; MDE, mid-domain effect; SAC, summed age of colonization; SE, standard errors; VIF, variance inflation fator.

**
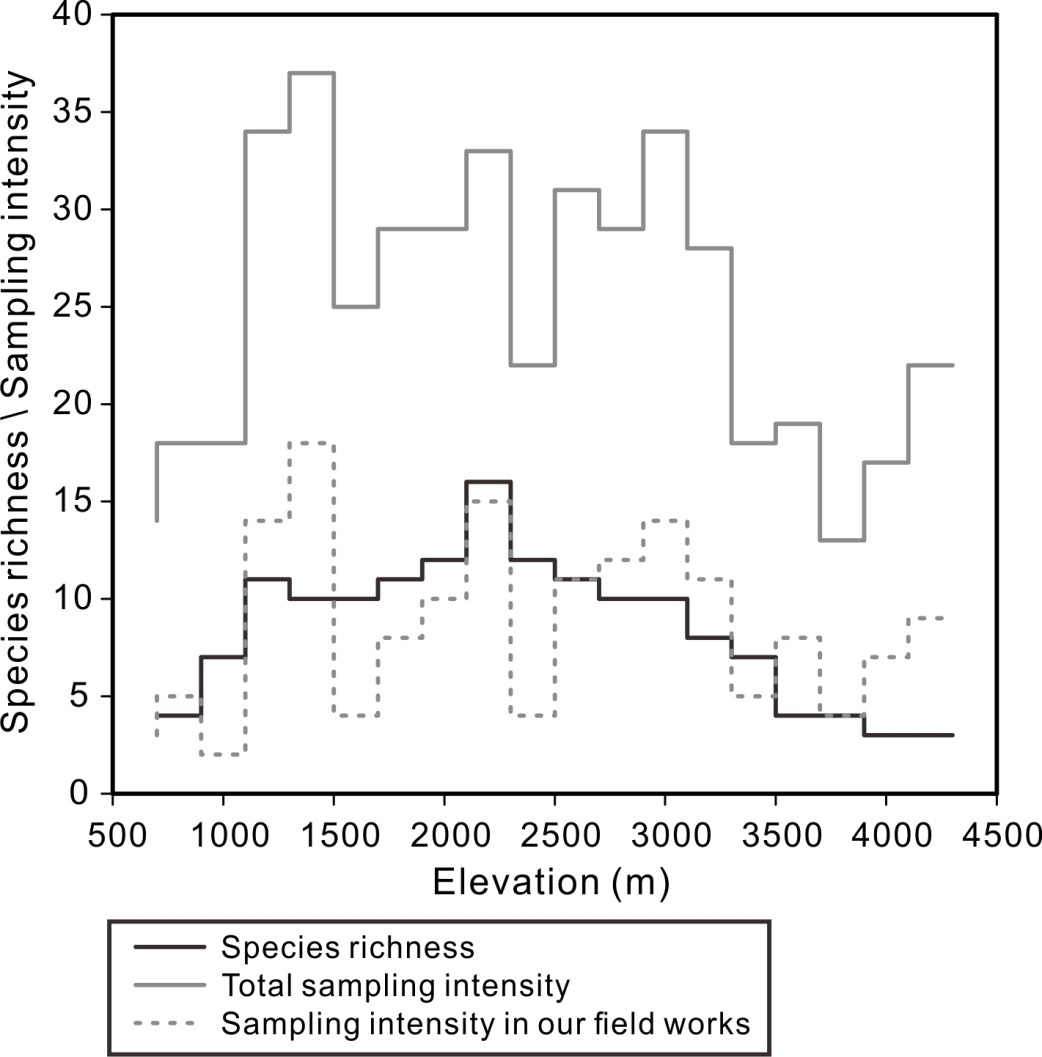
**

**Figure A1** Species richness pattern in the elevational gradients along the range from 600 to 4300 m. Black polygonal line is the result based on interpolation method; Gray polygonal line presents the total sampling intensity along elevation; Dotted line depicts sampling intensity in our field surveys along elevation.

**
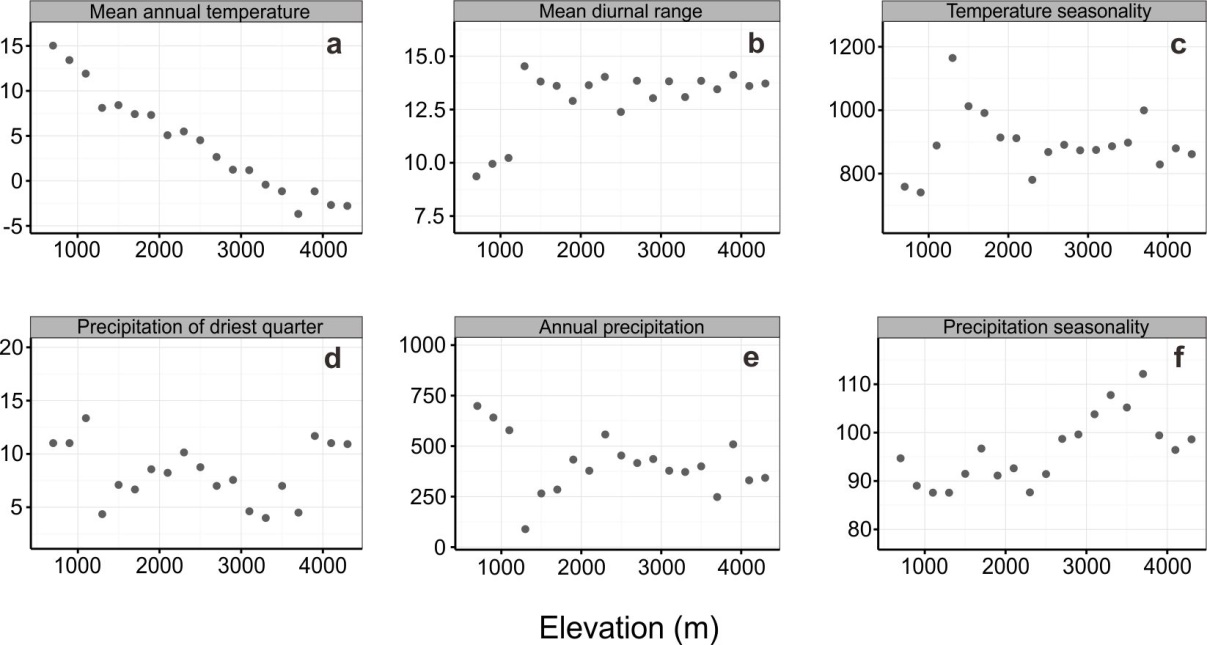
**

**Figure A2** Relationships between climatic variables and elevation. Panels (a - f) are labeled with descriptions at the top.

**
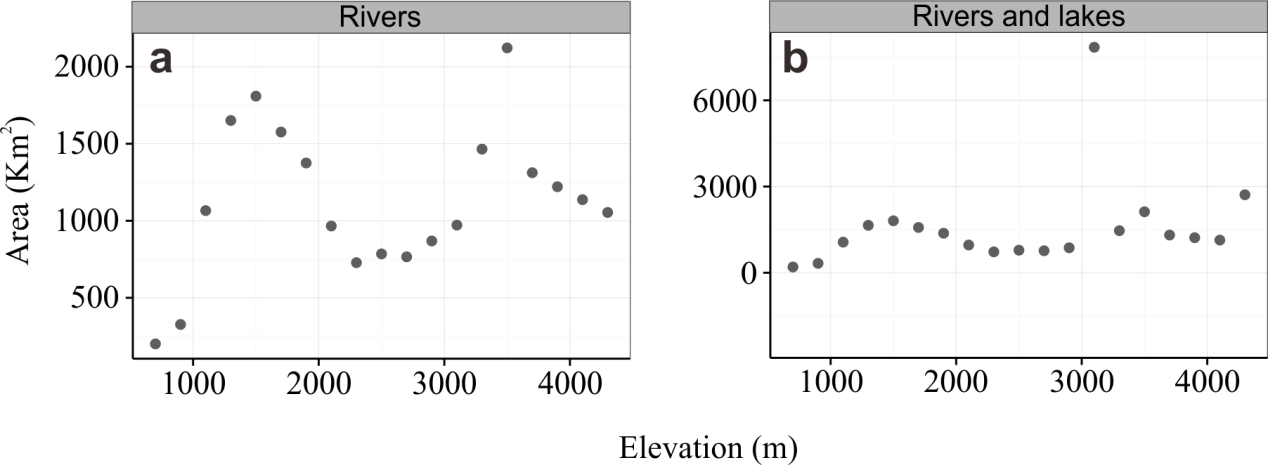
**

**Figure A3** Allocation of areal variables in each elevational band. We used (a) river basin area (rivers without lakes) and (b) total drainages area (rivers and lakes) to denote the area in each band respectively.

**
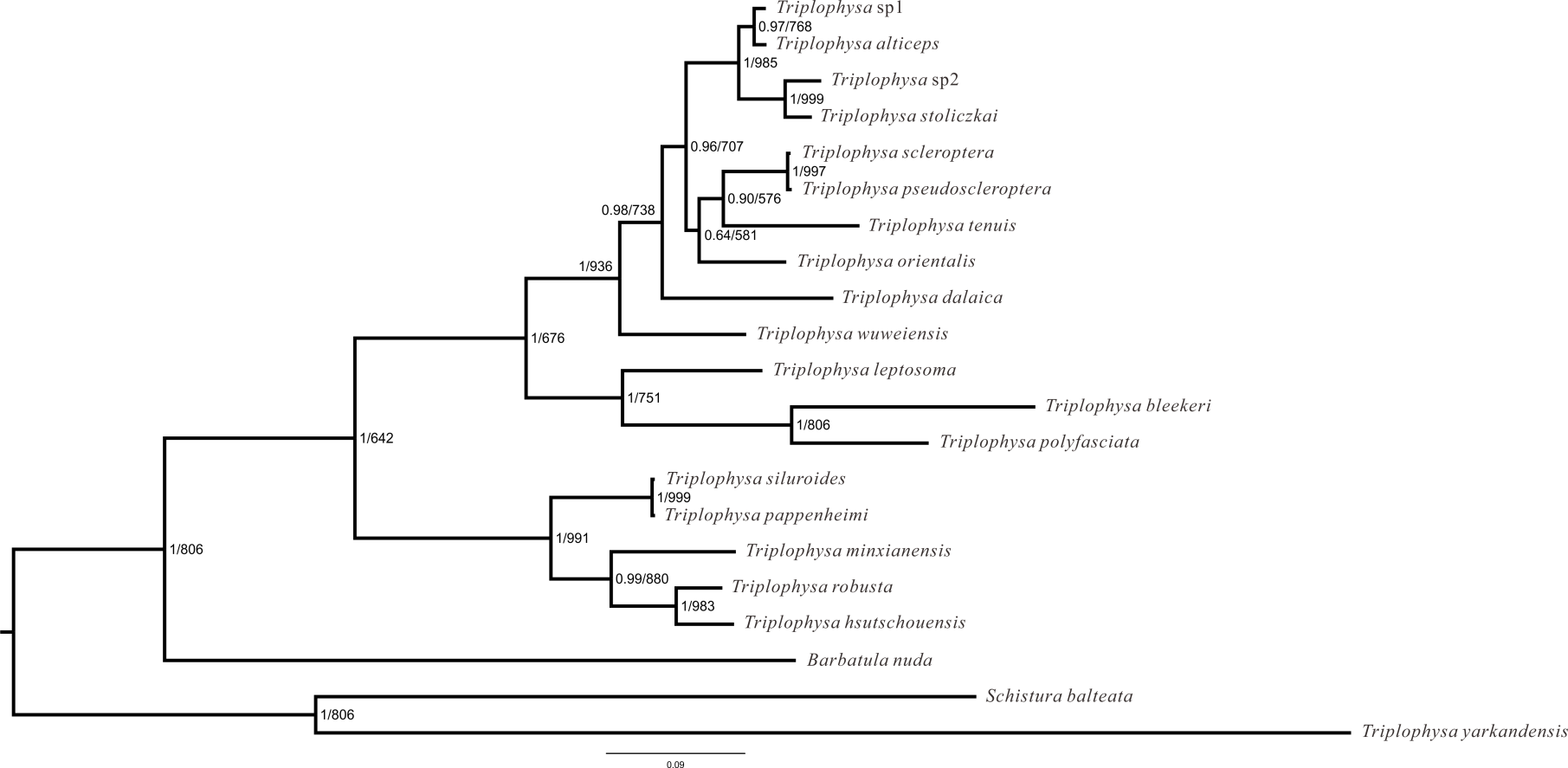
**

**Figure A4** Phylogram of the relationships within *Triplophysa* inferred from Bayesian analysis. Posterior probabilities (PP, left) obtained in the Bayesian analyses and bootstrap values (BS, right) obtained in the ML analyses are indicated on branches, respectively.

**
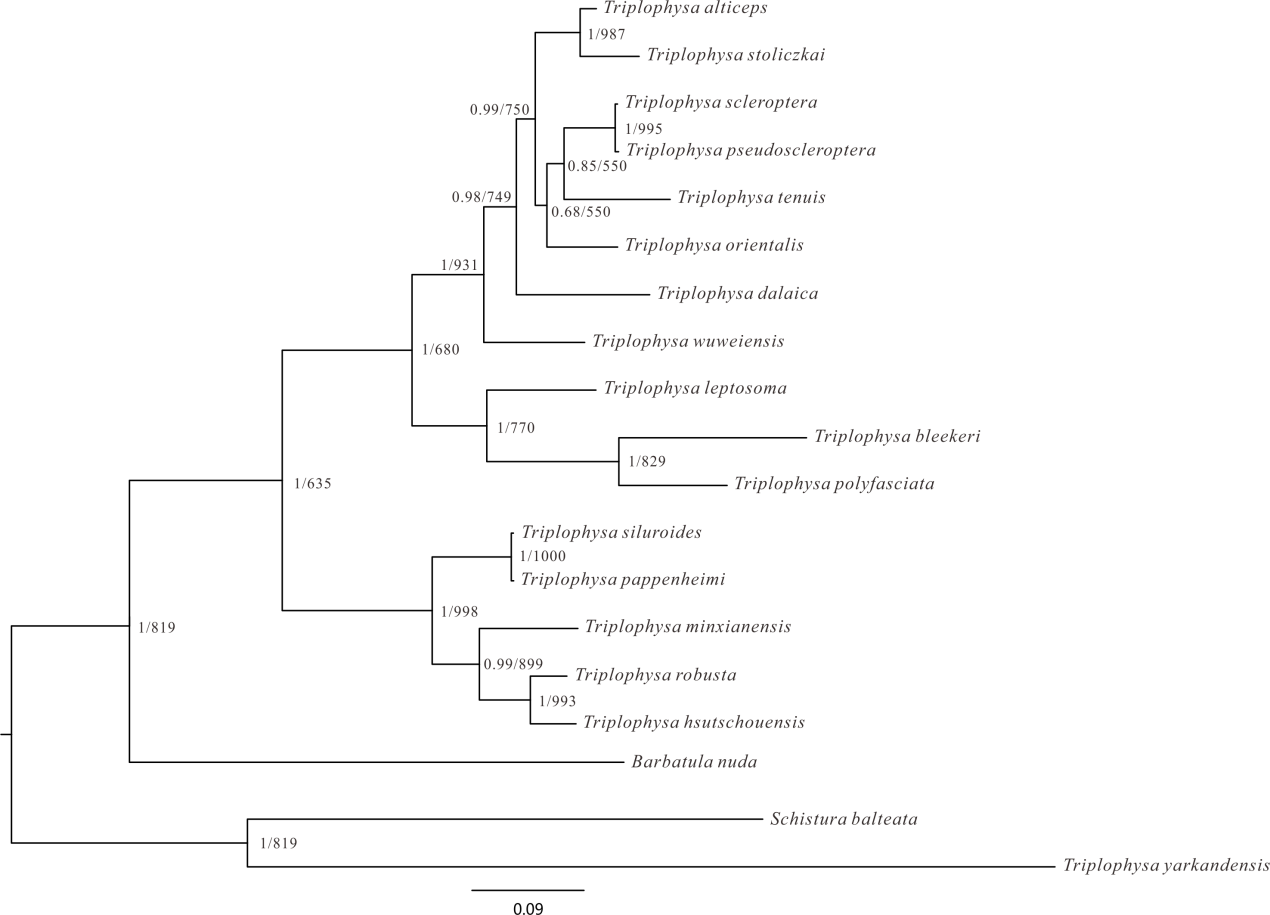
**

**Figure A5** Phylogram of the relationships within *Triplophysa* removing the two undescribed species inferred from Bayesian analysis, yielded unanimous topological trees with Figure S7 for the remaining species. PP (left) obtained in the Bayesian analyses and BS (right) obtained in the ML analyses are indicated on branches, respectively.

**
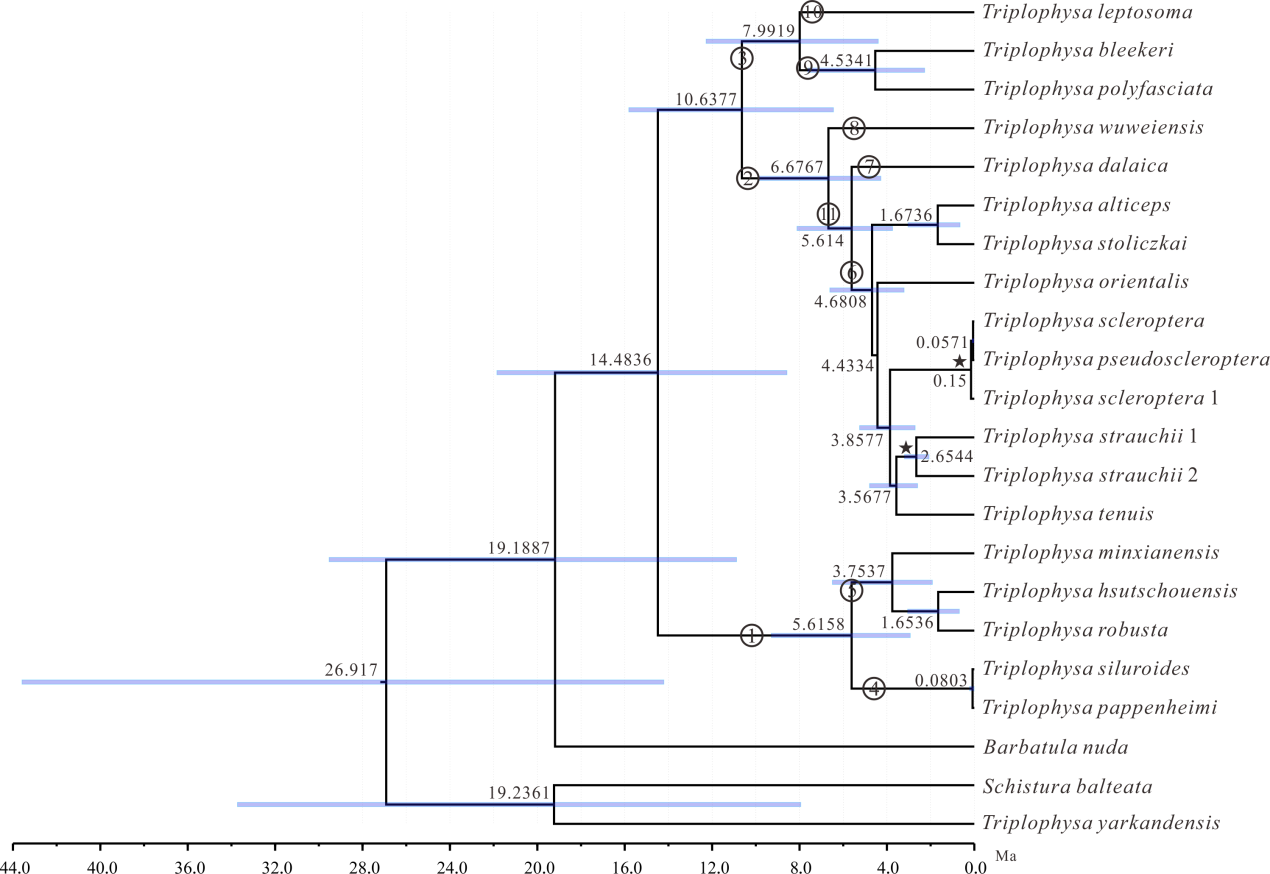
**

**Figure A6** BEAST chronogram of the *Triplophysa* inferred from two calibration points (black stars). Bars express 95% highest posterior density for nodes with a PP of >0.5. Numbers indicate estimated nodal ages in millions of years. The clade category used to estimate diversification rates is marked on the branch with number in circle.

**
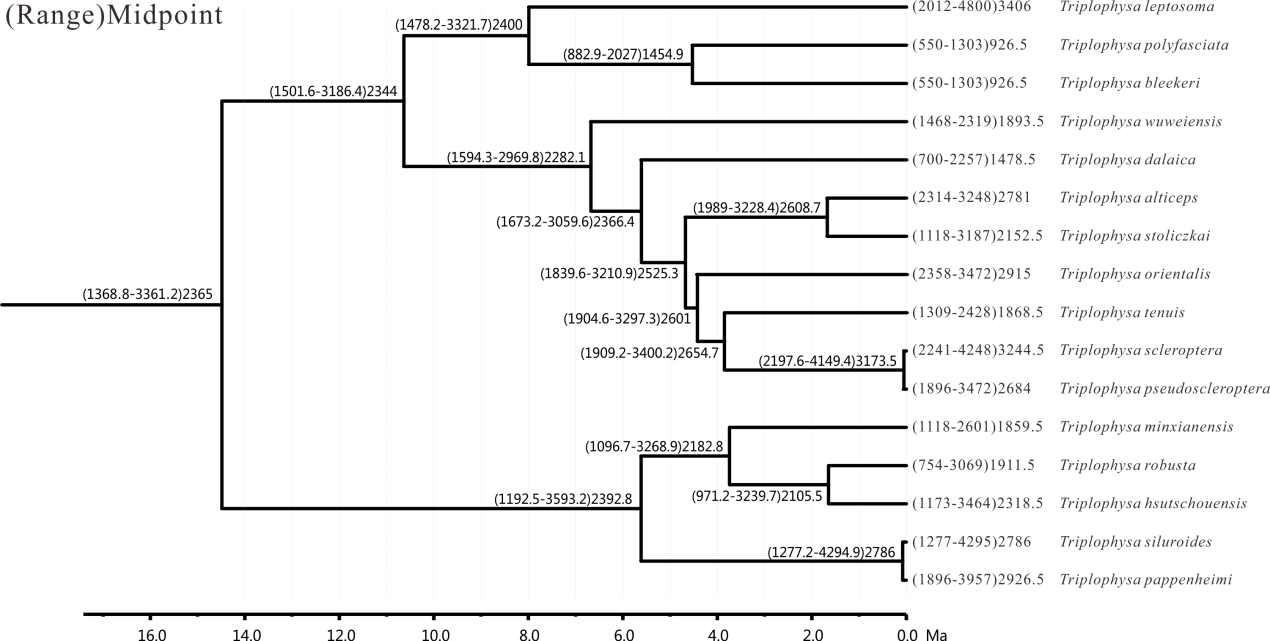
**

**Figure A7** Reconstructed ancestral states (range sizes and midpoints of distribution in elevation) of Tibetan loaches based on the tree obtained in the ML analyses. Values near the nodes are the estimated results for ancestors using parsimony ancestral states. Outgroup taxa are not shown.

**
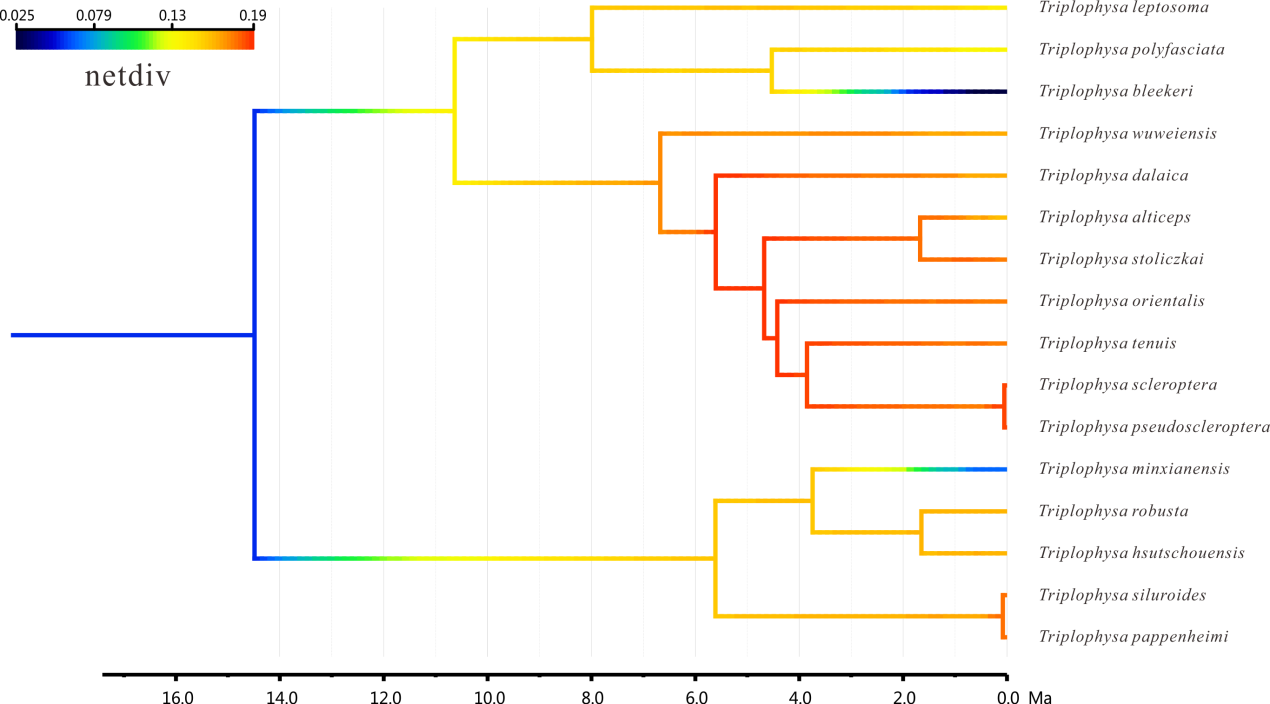
**

**Figure A8** ML phylogeny of Tibetan loaches, with BAMM estimates of instantaneous net diversification rate (netdiv) represented by colors along individual branches.

**
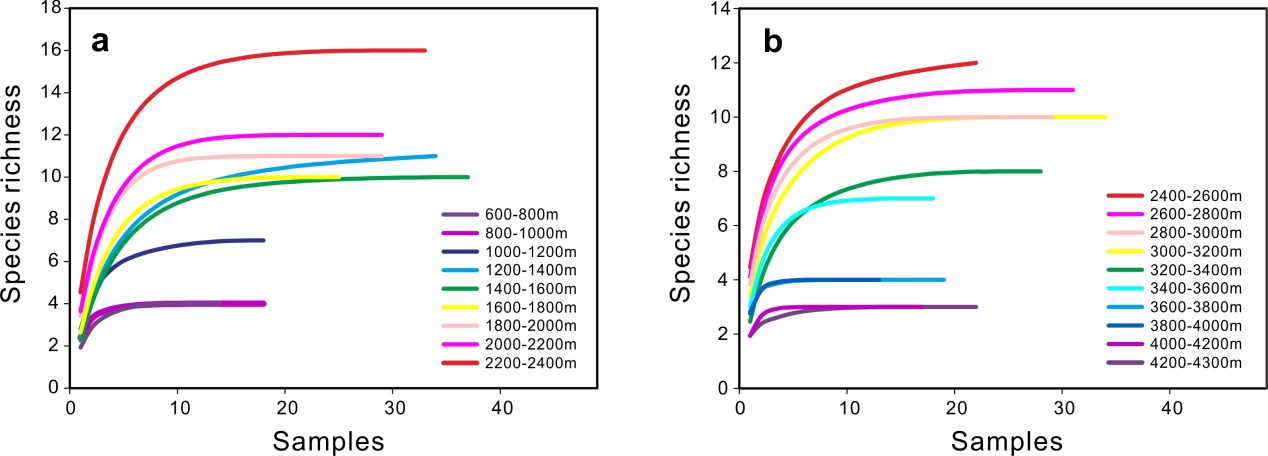
**

**Figure A9** Rarefaction analysis: species accumulation curves over samples. (a) 600-2400m; (b) 2400-4300m. The species accumulation curves assume adequate surveys of species.

**
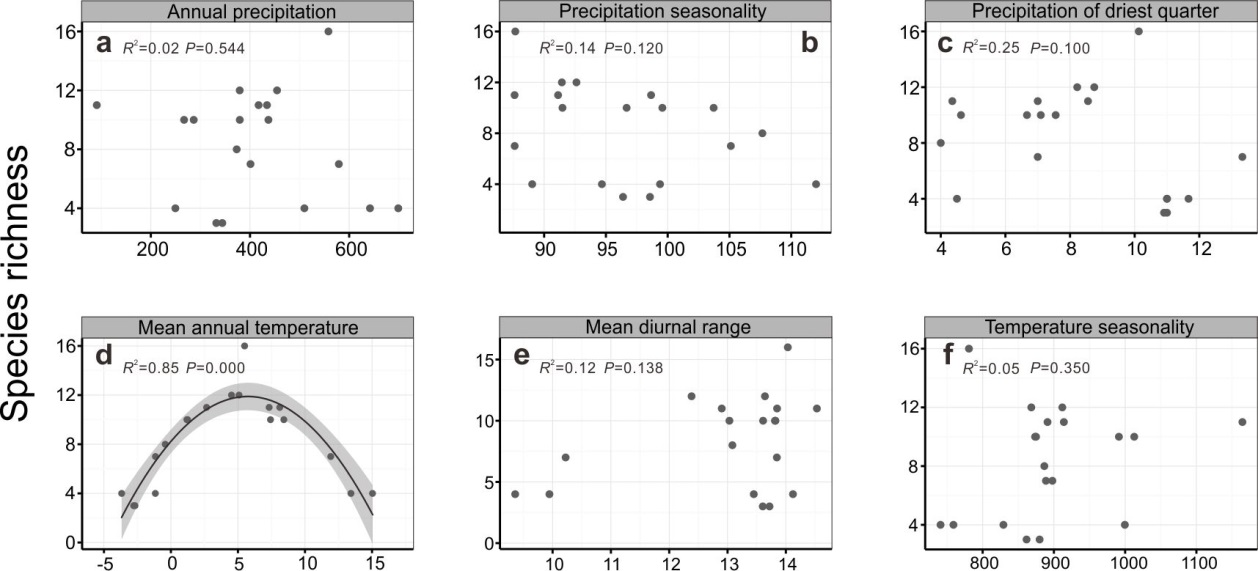
**

**Figure A10** Relationships between species richness and climatic factors. Panels (a - f) are labeled with descriptions at the top. Black fitted lines are from OLS regression. Shaded areas show 95% confidence interval of the fit. Only significant fitted lines are shown on the graphs.

**
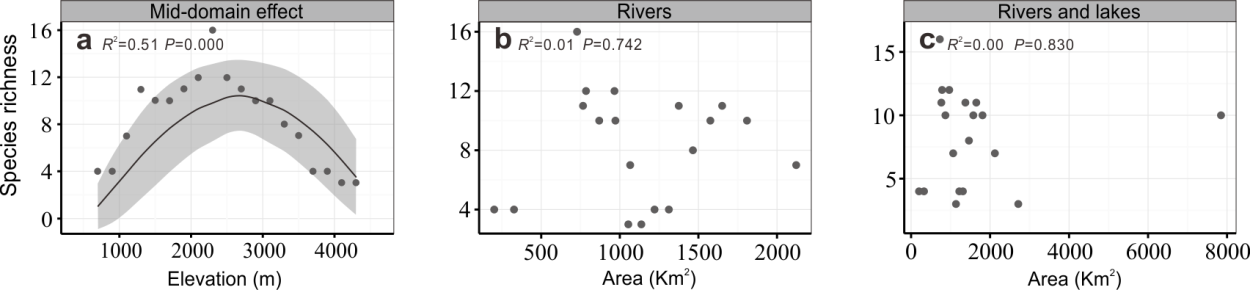
**

**Figure A11** Relationships between species richness and spatial factors. Results with non-significant statistical relationship are not shown on the graphs. (a) Relationship between mid-domain effect and species richness; dots represent the actual species richness, black curve is the predicted mean richness derived from RangeModel. Shaded areas show 95% confidence interval of the prediction. (b) Relationships between species richness and river basin area. (c) Relationships between species richness and total drainages area.

**
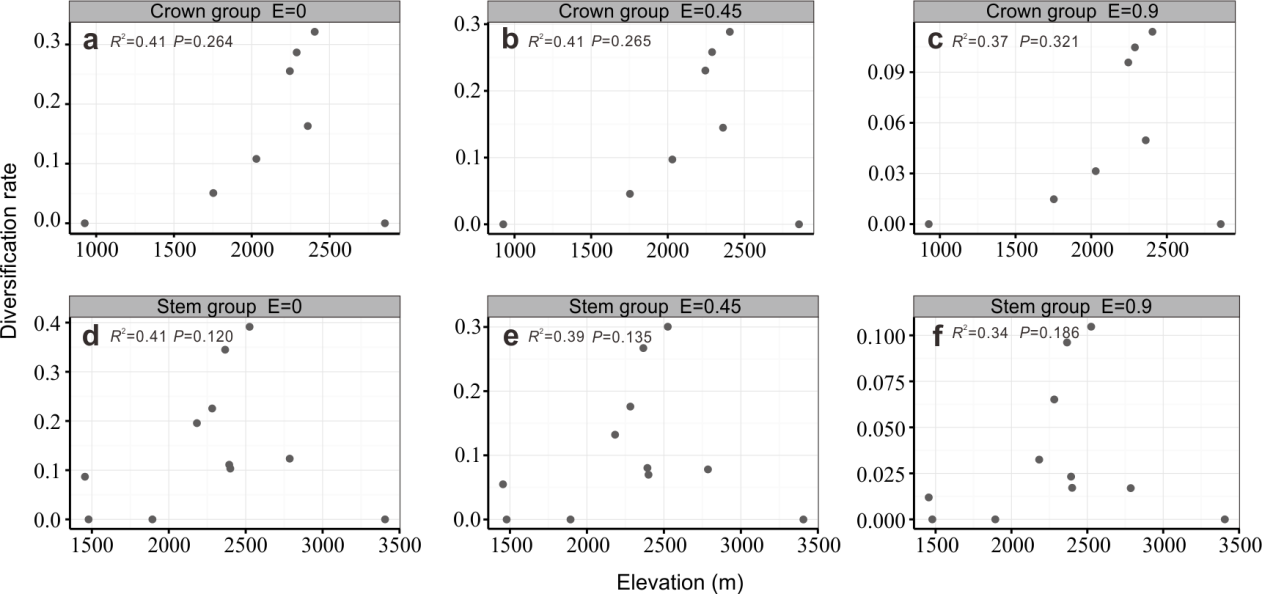
**

**Figure A12** Diversification rates in relation to elevation for the 11 clades, with the method-of-moments (Magallon and Sanderson 2001). The diversification rates are estimated using stem and crown group age based on three particular values of relative extinction rates (E=0, 0.45, 0.9). Panels (a - f) are labeled with descriptions at the top.

**
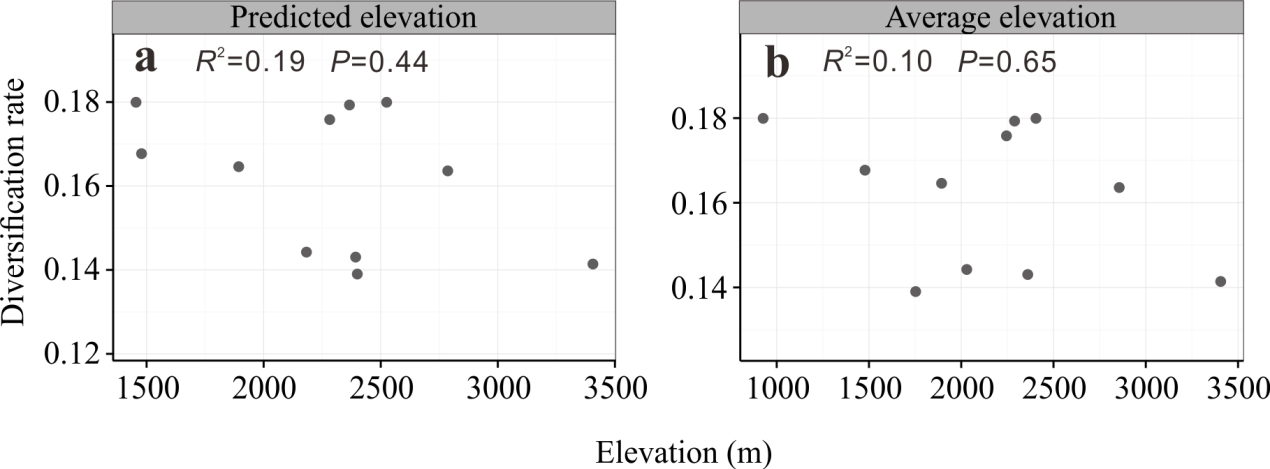
**

**Figure A13** Diversification rates in relation to elevation for the 11 clades, with the estimation under BAMM model (Rabosky et al., 2014). Predicted elevation (a) is the elevational midpoint of last common ancestor of each clade predicted by MESQUITE. Average elevation (b) is the average elevational midpoint of total species in a clade.

**
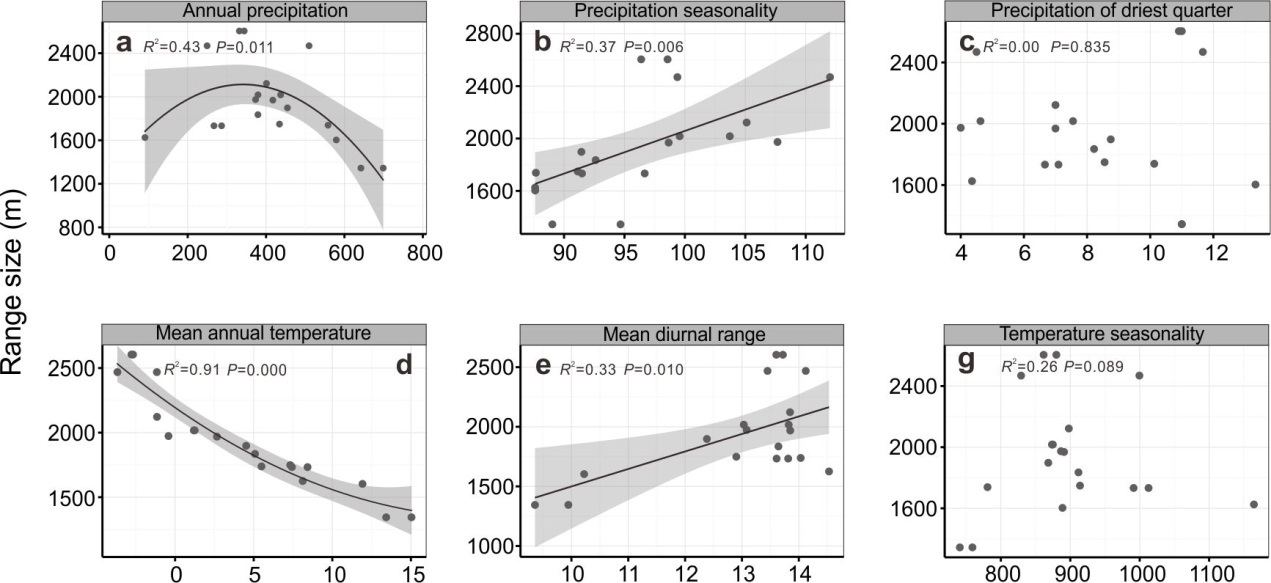
**

**Figure A14** Relationships between average range variation and climatic factors. Panels (a - f) are labeled with descriptions at the top. Black fitted lines are from OLS regression. Shaded areas show 95% confidence interval of the fit. Only significant fitted lines are shown on the graphs.

**References**

Magallon, S. & Sanderson, M.J. (2001). Absolute diversification rates in angiosperm clades. *Evolution*, 55, 1762-1780.

Rabosky, D.L., Donnellan, S.C., Grundler, M. & Lovette, I.J. (2014). Analysis and visualization of complex macroevolutionary dynamics: an example from Australian scincid lizards. *Systematic Biology*, 63, 610-627.

Saitoh, K., Sado, T., Mayden, R., Hanzawa, N., Nakamura, K., Nishida, M. & Miya, M. (2006). Mitogenomic evolution and interrelationships of the Cypriniformes (Actinopterygii: Ostariophysi): The first evidence toward resolution of higher-level relationships of the world’s largest freshwater fish clade based on 59 whole mitogenome sequences. *Journal of Molecular Evolution*, 63, 826-841.

Zhao, X., Hu, S., Xie, P., Ao, M., Cai, L., Niu, J. & Ma, X. (2015). The complete mitochondrial genome of Barbatula nuda (Cypriniformes: Nemacheilidae). *Mitochondrial DNA*, 26, 692-693.
